# New York Letter

**Published:** 1883-04

**Authors:** 


### Domestic Correspondence.

---

### ARTICLE XII.

EDITOR OF CHICAGO MEDICAL JOURNAL AND EXAMINER:

I have no doubt that the medical profession is somewhat tired of the subject of medical ethics. But the discussion continues, and recently the *N. Y. Medical Journal* has opened its columns to a series of articles pro and con. Perhaps I may be excused, therefore, for saying something of the matter now. The disinterested observer, who visits New York, would have to question many physicians before he found one who felt any particular interest in the present ethical fight. At the best attended meeting, called to vote upon the subject, only about one-fourth of the members of the County Society and perhaps one-tenth of the regular profession of the city was present. The general feeling is, that the real importance of the question has been exaggerated by outside agencies for an advertising purpose; and that medical practice here, as everywhere, goes on with very little reference to disciplinary codes. This view is strengthened by the fact that no case of discipline for consulting with irregulars has occurred in fifteen years in this city, or, I believe, in the State. It is somewhat curious that before the new code instances of sinful consultation by certain eminent physicians were well known to occur, and that some, to whom the practice was most ascribed, are now vigorously championing the old code.

It is a mistake to think that there is any especial antagonism among the physicians of the city. I do not think that many favor the present code as a whole, but only the now restrictive clause in it. The majority prefer, I should say the declaratory resolutions or Genterman's code. Many others would like the old code minus the restriction clause, and a little refurbished in other respects.

It is idle to expect that New York will ever return to the old code in its entirety, however. It would require a two-thirds vote to do it, and at the last meeting of the State Society, despite most laborious canvassing, there was not even a majority. The meeting was a full one, and each side did its best to bring out all its friends. The battle was fairly fought with no parliamentary tricks or political manipulation. The vote represented the general feeling in the State. This is shown by the canvas made by Dr. Smith, the secretary of the society. Among nearly 700 answers to his query as to which side the individuals took upon the matter of the code, there were about 340 in favor and not quite 300 against the present code. About one-third of the profession of the State live in New York, and less than one-third of the answers came from this city, the "ayes" and "noes" being about 80 to 120, as I remember the figures. So that the returns represent the feeling in the country, as well as city.

The statement is made that of the 59 county societies, only 6 have taken action in favor of the present code, while 36 have taken action against it. It should be borne in mind, however, that the six include New York county, Albany county and King's county, and that their representation is 47 out of the total of 123 county delegates. Assuming that the 17 counties which took no action had but one delegate, there would still be considerably over one-half of the county representation which took no action against the code. I have assumed that King's county is favorable to non-restriction, because I understand and believe that the vote rescinding instructions to delegates really represented the feeling of the majority of Brooklyn practitioners.

Perhaps one reason why the State of New York sustained its last year's action, is to be found in the character of the criticisms made upon it. Here are some examples :

"It disturbed 'a custom *approved and sanctified by wisdom and experience of ages.*'"

"A disgraceful act."

"In the interest of specialists."

"It (the Code) does not exclude the licensed cancer quack, midwife or chiropodist."

"Fifty doctors, reckless of honor, and greedy for gold, undertook to sell out the profession."

"A complete surrender to homœopathy."

"It (the code) asserts the propriety of consulting with homœopathists."

"Most unwise, ill-timed and injurious * * * untenable in every respect and not sustained by the action of any other respectable body in Europe or America."

"An unwise and useless code—a code which sacrifices the self-respect and honor of the members of a once honorable profession in order to pander to the interests of a few specialists."

"Hurried through by a small majority."

"A significant fact that the new code agitation was entirely inaugurated by specialists, and that every man who has taken at all an active part in securing its adoption and preventing its repeal is a specialist."

It has been keenly felt that these charges are, every one of them, untrue and unjust. Their constant iteration has, no doubt, made converts to the other side. For this reason, also, many in the city who did not entirely approve of the new code, felt gratified after all that it was sustained.

The charge against the specialists is made each time with as much triumph as if the authors had achieved an intellectual victory and satisfactorily solved the whole problem of ethics. But I think all candid men will agree that this is a side issue; that the question is really as to the right and advisability of allowing individual freedom in professional conduct, just as is allowed to members of all other professions and pursuits.

At any rate, in the State Society, it was not a queston between specialists and general practitioners, for among the 105 votes for the new code, I can count only 16 specialists. In this city the proportion of specialists among the anti-code men is relatively considerable. The Southern and Western gentlemen who say such severe things against the honesty of New York physicians, are probably ignorant of the methods in which specialists here do their work. This is chiefly done in the office, and the general practitioner does not bring his patients, but sends them. The patient is examined and prescribed for independently of his own physician.

I do not write to defend our specialists, however, who can, no doubt, take care of themselves; but only for fair treatment towards them and towards the profession of New York. It seems to me that there is no call for so much violent feeling and vituperative language. The profession is not going to be destroyed or disintegrated, as has been intimated. I do not think that we are victims of moral decay. I find it perfectly safe to mingle freely with my neighbors. The view in this city, I think, is, that by removing a restriction that had been a dead letter, we have placed ourselves in a better light before the world; have removed an affront and a stimulus to homœopathic progress, and have only set aside a technical morality which infringed on the individual's rights without elevating him in return. We believe that the true work for professional elevation lies in securing a higher educational standard, better trained minds, a more scientific spirit, and a greater technical skill in our midst. We believe that this work can be better done without the embarrassment and reproach caused by the restrictive code. We believe that skilled and learned physicians, obedient to the laws of God and the dictates of common morality are what the profession needs.

Why cannot the profession at large admit that we too in New York may, perhaps, be honest and earnest in our views. And is it not barely possible that the American Medical Association has been a little hasty in cutting off from its membership a State like New York, because of an honest disagreement in by-laws?

The Cartwright lectures, recently delivered by your townsman, Dr. W. T. Belfield, have excited much interest, and have been very well received. Dr. Belfield has done good service in showing that there is a genuine basis to the new bacterial pathology, and that micro-organisms cannot be dismissed, as is so often done, with a contemptuous reference to German theories.

The Post-Graduate School and the New York Policlinic have both done very well during the past season, and both have secured unquestioned success. With the benefits of a year's experience, no doubt there will be still further improvements in the future.

Our didactic colleges are beginning to be aroused to the fact that these post-graduate institutions mean something.

We are to have a new morgue, at an expense of $50,000. The present morgue is a mere shed, where bodies are piled away in coffins, awaiting inspection and identification, and mortuary ceremonies. There is a room with marble tables, separated from a passage-way by a glass partition, like that of the Paris morgue. The exposure of bodies here, however, drew so many persons, that it is not now used. There were 6000 corpses received at the morgue yearly, of whom about 160 were unidentified.

The hospital Saturday and Sunday collections fell off somewhat this year. The collection in 1881 was $42,000. In 1882-3 it was not quite $40,000. The reason, it is said, is that the churches were not attacked in the right way, that there was too much Episcopalianism in the committee, and too much reliance upon violent advertising in the paper. The plan is a good one, however, and it will doubtless be more successful next year. The Catholics here refuse to join the movement.

The profession has sustained a great loss in the sudden death of Dr. Beard. He was a tireless worker, and one of the most agreeable, witty and original of men. He left two works in manuscript, which will shortly be published. One was entitled "Medical Education," the other "Sexual and Other Varieties of Neurasthenia." The manuscripts were left by him in charge of Dr. Charles L. Dana, who will edit them.

One of the new, but most useful organizations in the city is the Charity Organization Society. One hundred and eight religious and charitable agencies are now making regular exchanges through it. The number of cases reported to the society up to December 1 was 37,937, while 4,557 notices concerning cases of duplicate relief have been sent to parties interested. There has been established a Bureau of Fraudulent Cases, and blanks have been sent to charitable institutions to be filled out with names of begging letter-writers, professional mendicants, hospital-rounders, and other impostors upon the charitable public.

Two of the colleges have just held their commencements. The University graduated a class of 164; Bellevue, a class of 166. It is said that both colleges are somewhat more rigid in examinations than formerly, and both, no doubt, do as much as is possible for a student in two six-month courses. The College of Physicians and Surgeons continues to be successful in sustaining its longer term.

The subject of convallaria maialis has been much talked of in medical societies of late. On the whole, the conclusion is being reached that it is less valuable than digitalis. It seems to be especially efficacious in relieving dyspnœa, due to emphysema or a weakened heart. In a case of this kind of my own, its effects were good, but very temporary.

Efforts are being made to obtain a law taking the power of licensing away from the colleges and giving it to a State Board of Examiners. The plan was endorsed by the State Society, and it is said to have a good chance of being carried out. The colleges of this city do not oppose it, at least two of them do not. The law, if enacted, will also modify and improve somewhat the registration law. This latter has worked well in New York City, but it has been a dead letter, so far as the prosecution of those fraudulently registering is concerned, in other parts of the State. It is claimed that medical law-making has been a failure, but though there have been mistakes, the charge, as a whole, is hardly true. It is believed that by suitable laws we can secure a system of licensing and registering which will exclude quacks and greatly elevate the general status of the doctors throughout the State.

M. S.

NEW YORK, March 20, 1883.

---

MORTALITY, CHICAGO, MONTH OF FEBRUARY, 1883.—Dr. DeWolf, Health Commissioner, reports during February a total mortality of 859. This is 119 less than that for February of 1882. Pneumonia is the first disease in number of deaths (87); infantile convulsions (80) is next; phthisis pulmonalis (79) next; diphtheria (34) next; murder, two; manslaughter, two.

